# Multimodality imaging features of dasatinib-induced pulmonary arterial hypertension

**DOI:** 10.1016/j.jccase.2026.07.019

**Published:** 2026-08-18

**Authors:** Chikako Awazu, Seitaro Oda, Seika Furuie, Ryo Shinohara, Hidetaka Hayashi, Masafumi Kidoh, Kyoko Hirakawa, Naoto Kuyama, Eiichiro Yamamoto, Yasuhiro Izumiya, Kenichi Tsujita, Toshinori Hirai

**Affiliations:** a Department of Diagnostic Radiology, Faculty of Life Sciences, Kumamoto University, Kumamoto, Japan; b Department of Cardiovascular Medicine, Graduate School of Medical Sciences, Kumamoto University, Kumamoto, Japan

**Keywords:** Pulmonary hypertension, Dasatinib, Computed tomography, Cardiac magnetic resonance

## Abstract

Dasatinib, a tyrosine kinase inhibitor used to treat chronic myelogenous leukemia, is associated with drug-induced pulmonary arterial hypertension (PAH). We report two cases of dasatinib-induced PAH evaluated using advanced noninvasive multimodality imaging. A comprehensive pulmonary hypertension-targeted computed tomography (CT) protocol showed no structural lung abnormalities or pulmonary embolism. However, CT pulmonary perfusion imaging revealed diffuse heterogeneous hypoperfusion in both lungs, suggesting functional pulmonary circulatory impairment. Myocardial delayed-enhancement CT demonstrated late iodine enhancement and increased extracellular volume at the right ventricular insertion points, indicating myocardial involvement related to right ventricular pressure overload. Cardiac magnetic resonance confirmed right ventricular remodeling, and right heart catheterization (RHC) established precapillary pulmonary hypertension. After discontinuation of dasatinib and initiation of pulmonary vasodilator therapy, one patient achieved partial remission and the other normalization of pulmonary hemodynamics on follow-up RHC. These findings underscore the clinical value of advanced imaging in diagnosing and characterizing drug-induced PAH.

**Learning objective:**

To understand the clinical significance of noninvasive multimodality imaging, including a comprehensive pulmonary hypertension-targeted computed tomography protocol, in dasatinib-induced pulmonary arterial hypertension and to integrate imaging findings for a systematic assessment of its pathophysiology and clinical presentation.

## Introduction

Pulmonary hypertension (PH) is a heterogeneous group of progressive disorders characterized by increased pulmonary vascular resistance leading to right ventricular overload, with exertional dyspnea and fatigue as common symptoms [1]. Hemodynamically, PH is defined as a mean pulmonary arterial pressure (mPAP) >20 mmHg at rest on right heart catheterization (RHC) and is classified into five groups according to etiology [1], [2]. Noninvasive multimodality imaging plays a central role in diagnosis and severity assessment, beginning with transthoracic echocardiography (TTE) for screening, followed by structural and etiological evaluation using computed tomography (CT) and ventilation–perfusion scintigraphy, as well as assessment of right ventricular morphology and function with cardiac magnetic resonance imaging (CMR) [3]. This integrated approach supports risk stratification and therapeutic decision-making.

Drug-induced pulmonary arterial hypertension (PAH) results from medication-related pulmonary vascular endothelial injury and remodeling, leading to precapillary PH. Dasatinib, a tyrosine kinase inhibitor used to treat chronic myelogenous leukemia, is a well-recognized cause of PAH [4]. We report two cases of dasatinib-induced PAH evaluated using advanced noninvasive multimodality imaging and review the relevant literature.

## Case report

### Case 1

A woman aged in her 50s had been diagnosed with chronic myeloid leukemia two years earlier and treated with dasatinib. During follow-up, exertional dyspnea developed, and PH was suspected on TTE. Although dasatinib was discontinued, PH persisted and she was referred to our institution. Laboratory testing showed elevated B-type natriuretic peptide (137.6 pg/mL) and liver dysfunction consistent with congestive hepatopathy. On electrocardiography, sinus rhythm at 81 beats/min was observed, with a strain pattern in leads V1–V3 and P pulmonale in lead II, suggesting right ventricular overload. TTE revealed right ventricular enlargement and septal flattening, with a tricuspid regurgitation pressure gradient of 57 mmHg and estimated pulmonary artery systolic pressure of 61 mmHg; left ventricular wall motion was preserved. A comprehensive PH-targeted CT protocol demonstrated no parenchymal lung abnormalities or pulmonary embolism. However, visual assessment of pulmonary perfusion CT revealed diffuse heterogeneous hypoperfusion in both lungs. Myocardial delayed-enhancement CT showed D-shaped remodeling and late iodine enhancement (LIE) with increased extracellular volume (ECV) fraction at the right ventricular insertion points, indicating myocardial involvement due to right ventricular pressure overload. Ventilation–perfusion scintigraphy showed no significant mismatch (Fig. 1). CMR demonstrated right ventricular dilation with reduced ejection fraction (34%), septal flattening, prolonged native T1, increased ECV, focal T2 prolongation, and late gadolinium enhancement at the right ventricular insertion points (Fig. 2). RHC confirmed precapillary PH (mPAP 42 mmHg, pulmonary capillary wedge pressure 4 mmHg, pulmonary vascular resistance 13 Wood units). Based on imaging findings and clinical history, Group 1 PAH associated with dasatinib was diagnosed. Pulmonary vasodilator therapy was initiated, and follow-up catheterization at 3 months showed improvement (mPAP 34 mmHg, pulmonary vascular resistance 5 Wood units), consistent with partial remission.

**Fig. 1:**
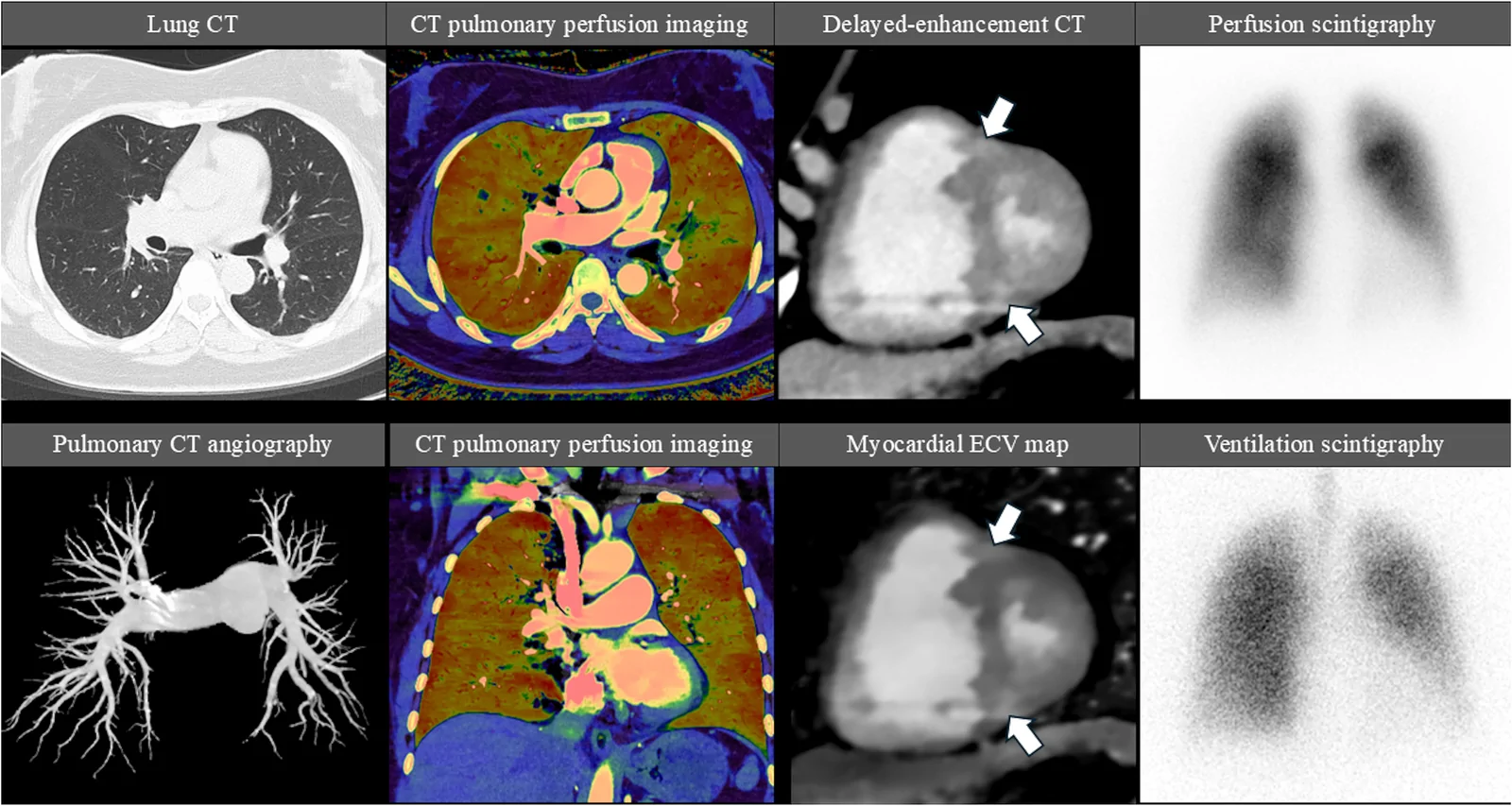
Comprehensive pulmonary hypertension-targeted computed tomography (CT), lung CT, and CT pulmonary angiography demonstrated no apparent parenchymal abnormalities or findings suggestive of pulmonary embolism. CT pulmonary perfusion imaging revealed diffuse and heterogeneous hypoperfusion in both lungs. On delayed-enhancement cardiac CT, right ventricular enlargement and septal flattening were observed. In addition, late iodine enhancement and increased extracellular volume (ECV) were identified at the right ventricular insertion points (arrows). Ventilation–perfusion scintigraphy showed no significant mismatch.

**Fig. 2:**
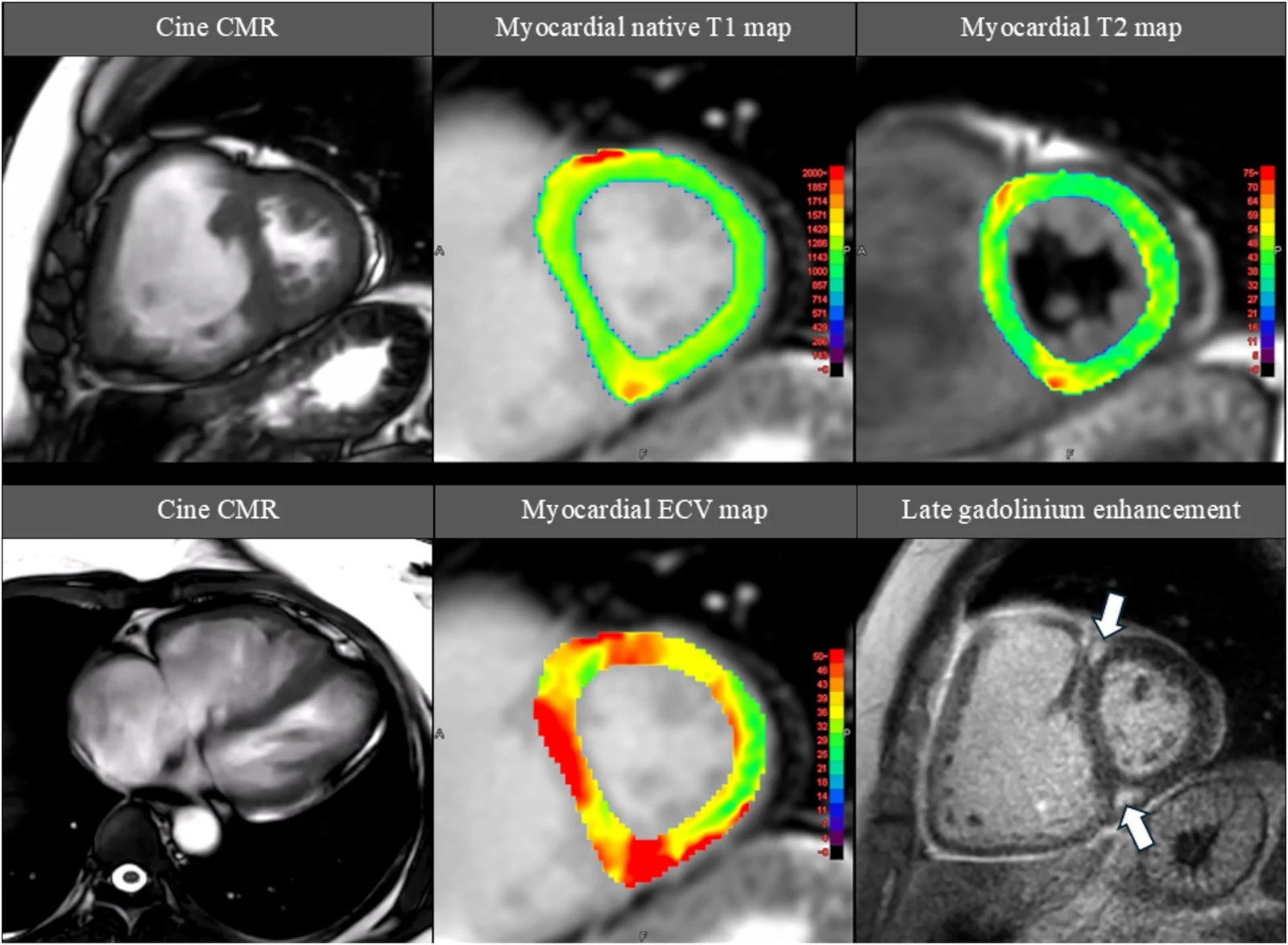
Cardiac magnetic resonance imaging (CMR) demonstrated right ventricular dilation with reduced ejection fraction (34%), septal flattening, prolonged native T1, increased extracellular volume (ECV), focal T2 prolongation, and late gadolinium enhancement at the right ventricular insertion points (arrows).

### Case 2

A woman aged in her 40s had been diagnosed with chronic myeloid leukemia six years earlier and treated with dasatinib. Exertional dyspnea developed one month before presentation and progressively worsened. TTE suggested PH, prompting discontinuation of dasatinib and referral to our institution. Laboratory tests showed mild hepatic dysfunction and renal impairment. Electrocardiography showed sinus rhythm (84 beats/min) with a QS pattern in V1–V2 and low limb‑lead voltage, raising suspicion of right ventricular involvement. TTE revealed right ventricular enlargement and dysfunction, with a tricuspid regurgitation pressure gradient of 62 mmHg and estimated pulmonary artery systolic pressure of 65 mmHg. A comprehensive PH-targeted CT protocol demonstrated pleural effusion and cardiomegaly without structural lung abnormalities or pulmonary embolism. Pulmonary perfusion CT revealed diffuse heterogeneous hypoperfusion in both lungs. Myocardial delayed-enhancement CT showed D-shaped remodeling with LIE and increased ECV at the right ventricular insertion points, indicating myocardial involvement related to right ventricular pressure overload (Fig. 3). RHC confirmed precapillary PH (mPAP 48 mmHg, pulmonary capillary wedge pressure 7 mmHg, pulmonary vascular resistance 18 Wood units). Based on clinical history and imaging findings, Group 1 PAH associated with dasatinib was diagnosed. After initiation of pulmonary vasodilator therapy, the mPAP decreased to 11 mmHg, with a pulmonary capillary wedge pressure of 5 mmHg and a pulmonary vascular resistance of 1.2 Wood units, all within the normal range.

**Fig. 3:**
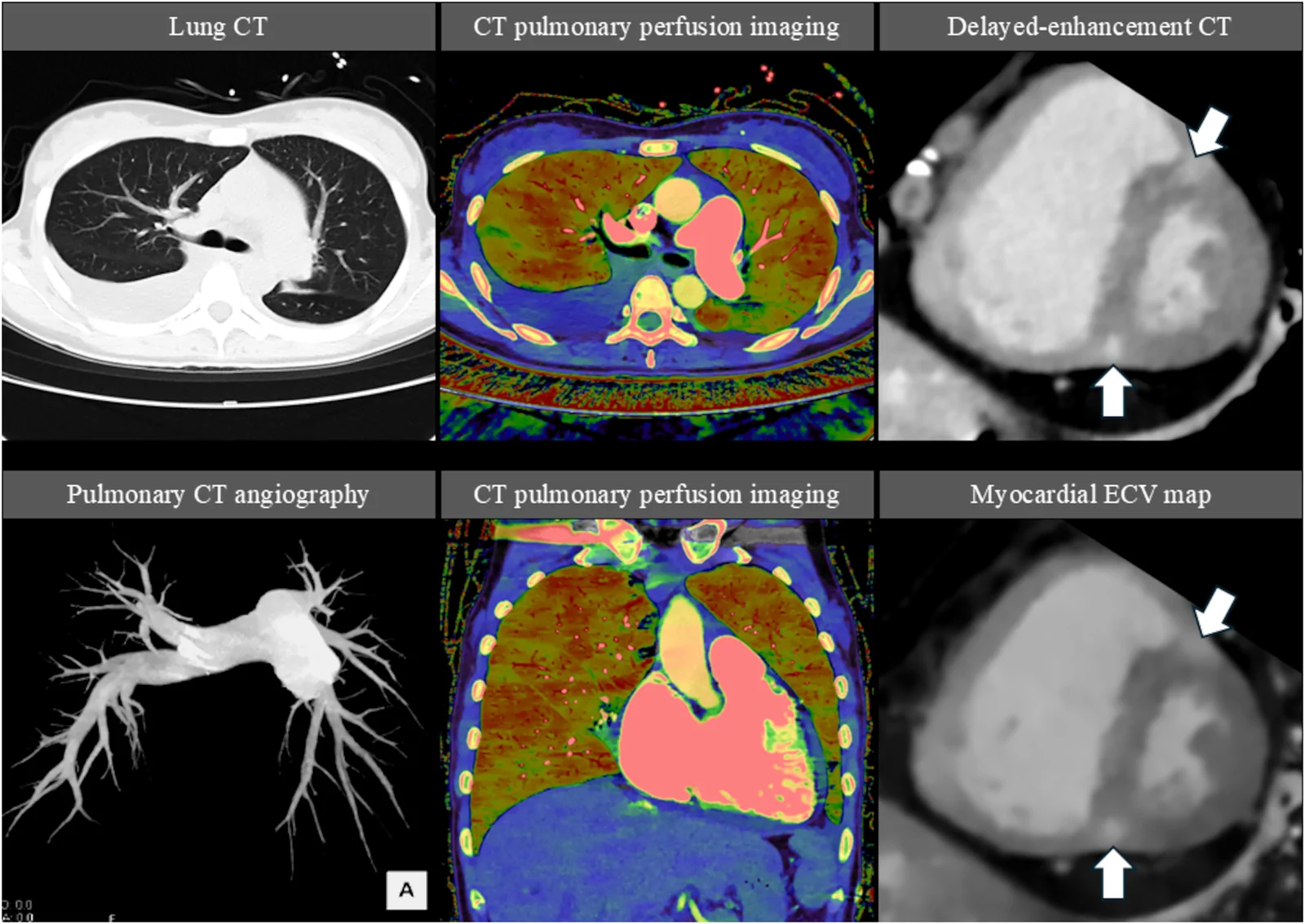
Comprehensive pulmonary hypertension-targeted computed tomography (CT), lung CT, and CT pulmonary angiography demonstrated no apparent parenchymal abnormalities or findings suggestive of pulmonary embolism; however, pleural effusion was observed. CT pulmonary perfusion imaging revealed diffuse and heterogeneous hypoperfusion in both lungs. On delayed-enhancement cardiac CT, right ventricular enlargement and septal flattening were noted. In addition, late iodine enhancement and increased extracellular volume (ECV) were identified at the right ventricular insertion points (arrows).

## Discussion

This report describes two cases of drug-induced PAH presumed to be attributable to dasatinib. In both patients, a comprehensive PH-targeted CT protocol revealed no structural lung abnormalities or pulmonary embolism, whereas pulmonary perfusion CT demonstrated diffuse heterogeneous hypoperfusion. Myocardial delayed-enhancement CT showed LIE and increased ECV at the right ventricular insertion points, consistent with myocardial involvement related to right ventricular pressure overload. RHC confirmed precapillary PH in both cases. Following pulmonary vasodilator therapy, one patient achieved partial remission and the other normalization of pulmonary hemodynamics on follow-up RHC. To our knowledge, this is one of the first reports to comprehensively describe advanced multimodality noninvasive imaging findings in drug-induced PAH. On the other hand, these imaging findings are not specific to drug-induced PAH and may also be observed in other forms of Group 1 PAH, including idiopathic PAH. Therefore, an integrated interpretation of imaging findings and clinical information is essential for the diagnosis of drug-induced PAH.

Dasatinib-induced PAH is classified as Group 1 PAH and may develop months to years after treatment initiation, without a clear relationship to cumulative dose [5]. Proposed mechanisms include endothelial dysfunction and apoptosis mediated by increased reactive oxygen species (ROS) [6]. Although discontinuation of dasatinib often improves hemodynamics, persistent PH has been reported, highlighting the importance of early detection and surveillance [6]. In our cases, treatment response varied, indicating interindividual differences in reversibility.

Imatinib inhibits platelet-derived growth factor receptor (PDGFR) in addition to breakpoint cluster region-Abelson (BCR-ABL) fusion kinase and has therefore been investigated as a potential therapeutic agent for PAH, because it may suppress pulmonary vascular smooth muscle cell proliferation and vascular remodeling mediated by PDGFR signaling. In contrast, dasatinib has a broader kinase inhibition profile, targeting BCR-ABL, c-KIT, and PDGFR, as well as SRC family kinases. These broad off-target effects have been reported to contribute to the development of PAH by inducing pulmonary vascular endothelial injury, increased ROS production, enhanced endothelial permeability, endothelial cell apoptosis, and abnormalities in pulmonary vascular tone.

In Group 1 PAH, TTE is useful for screening by demonstrating right ventricular enlargement, increased tricuspid regurgitation velocity, and septal flattening. CT typically shows main pulmonary artery enlargement and right heart dilation, whereas CMR provides detailed evaluation of right ventricular remodeling and myocardial abnormalities, including insertion point enhancement and T1 mapping changes [7].

The comprehensive PH-targeted CT protocol functions as a “one-stop shop,” enabling integrated evaluation of pulmonary structure, perfusion, cardiac morphology, and myocardial injury within a single examination [8]. By combining pulmonary CT angiography, perfusion imaging, and equilibrium-phase cardiac CT for LIE and ECV quantification, this approach allows simultaneous assessment of morphological and functional abnormalities and supports disease characterization and therapeutic decision-making.

Perfusion imaging plays an important role in excluding Group 4 PH, including chronic thromboembolic pulmonary hypertension (CTEPH). The diffuse heterogeneous hypoperfusion observed in the present cases was clearly different from the typical findings of CTEPH, which are characterized mainly by well-defined segmental or subsegmental mismatched perfusion defects. This distinction is an important diagnostic point. An inhomogeneous patchy pulmonary hypoperfusion pattern in PAH has been reported on pulmonary perfusion scintigraphy and CT, and has been found to be associated with disease severity and poor prognosis [9], [10]. However, CT pulmonary perfusion imaging and ventilation-perfusion scintigraphy differ substantially in spatial resolution. In addition, the methods of image display also differ, including iodine-density-based CT perfusion maps and relative radiotracer distribution on scintigraphy; therefore, the findings do not necessarily correspond completely between these modalities. For the assessment of diffuse pulmonary hypoperfusion in PAH, CT pulmonary perfusion imaging, with its higher spatial resolution, may be more sensitive. The assessment of CT pulmonary perfusion imaging in the present cases was based on visual evaluation, and the establishment of quantitative assessment methods is desirable for more accurate diagnosis.

In the present protocol, the contrast medium dose was 550 mgI/kg. The radiation exposure, expressed as CTDI_vol_, was 7.9 mGy for non-contrast CT, 8.9 mGy for the first contrast-enhanced phase, including pulmonary CT angiography and perfusion imaging, and 21.3 mGy for equilibrium-phase cardiac CT. Because patients with PH may have renal dysfunction or hepatic dysfunction, including congestive hepatopathy, the potential impact of contrast load should be carefully considered. RHC remains the gold standard for the definitive diagnosis, hemodynamic assessment, and severity evaluation of PAH. Advanced noninvasive imaging complements, but does not replace, RHC.

In conclusion, dasatinib-induced PAH may exhibit characteristic pulmonary perfusion and myocardial abnormalities on advanced imaging. The integrated use of a comprehensive PH-targeted CT protocol with multimodality imaging enhances diagnostic accuracy and facilitates detailed phenotypic assessment in drug-induced PAH.

## Consent statement

Informed consent was obtained from the patients for publication of this case report, including the accompanying images.

## Declaration of competing interest

This work was supported by the Japan Agency for Medical Research and Development (AMED) under Grant Number JP256f0137011.
